# Porcine Sapovirus-Induced Tight Junction Dissociation via Activation of the RhoA/ROCK/MLC Signaling Pathway

**DOI:** 10.1128/JVI.00051-21

**Published:** 2021-05-10

**Authors:** Muhammad Sharif, Yeong-Bin Baek, Ahsan Naveed, Nattan Stalin, Mun-Il Kang, Sang-Ik Park, Mahmoud Soliman, Kyoung-Oh Cho

**Affiliations:** a Laboratory of Veterinary Pathology, College of Veterinary Medicine, Chonnam National University, Gwangju, Republic of Korea; b Department of Pathology and Clinical Pathology, Faculty of Veterinary Medicine, Assiut University, Assiut, Egypt; Instituto de Biotecnologia/UNAM

**Keywords:** porcine sapovirus, tight junction, dissociation, RhoA/ROCK/MLC signaling pathway, virus entry

## Abstract

Tight junctions (TJs) are a major barrier and also an important portal of entry for different pathogens. Porcine sapovirus (PSaV) induces early disruption of the TJ integrity of polarized LLC-PK cells, allowing it to bind to the buried occludin coreceptors hidden beneath the TJs on the basolateral surface. However, the signaling pathways involved in the PSaV-induced TJ dissociation are not yet known. Here, we found that the RhoA/Rho-associated protein kinase (ROCK)/myosin light chain (MLC) signaling pathway was activated in polarized LLC-PK cells during the early infection of PSaV Cowden strain in the presence of bile acid. Specific inhibitors of RhoA, ROCK, and MLC restored PSaV-induced reduction of transepithelial resistance, increase of paracellular flux, intracellular translocation of occludin, and lateral membrane lipid diffusion. Moreover, each inhibitor significantly reduced PSaV replication, as evidenced by reductions in viral protein synthesis, genome copy number, and progeny viruses. The protein kinase C (PKC)/myosin light chain kinase (MLCK) and RhoA/ROCK/MLC phosphatase (MYPT) signaling pathways, which are known to dissociate TJs, were not activated during early PSaV infection. Among the aforementioned signaling pathways, the RhoA/ROCK/MLC signaling pathway was only activated by PSaV in the absence of bile acid, and specific inhibitors of this signaling pathway restored early TJ dissociation. Our findings demonstrate that PSaV binding to cell surface receptors activates the RhoA/ROCK/MLC signaling pathway, which in turn disrupts TJ integrity via contraction of the actomyosin ring. Our study contributes to understanding how PSaV enters the cells and will aid in developing efficient and affordable therapies against PSaV and other calicivirus infections.

**IMPORTANCE** PSaV, one of the most important enteric pathogens, is known to disrupt TJ integrity to expose its buried coreceptor occludin in polarized LLC-PK cells. However, the cellular signaling pathways that facilitate TJ dissociation are not yet completely understood. Here, we demonstrate that early infection with PSaV in polarized LLC-PK cells in either the presence or absence of bile acids activates the RhoA/ROCK/MLC signaling pathway, whose inhibitors reverse the early PSaV infection-induced dissociation of TJs and reduce PSaV replication. However, early PSaV infection did not activate the PKC/MLCK and RhoA/ROCK/MYPT signaling pathways, which are also known to dissociate TJs. This study provides a better understanding of the mechanism involved in early PSaV infection-induced disruption of TJs, which is important for controlling or preventing PSaV and other calicivirus infections.

## Abstract (precis)

PSaV, one of the most important enteric pathogens, is known to disrupt TJ integrity to expose its buried coreceptor occludin in polarized LLC-PK cells. However, the cellular signaling pathways that facilitate TJ dissociation are not yet completely understood.

## INTRODUCTION

Caliciviruses, which belong to the family *Caliciviridae*, are small (27 to 40 nm) nonenveloped viruses containing a single-stranded, positive-sense RNA genome of 7 to 8 kb (1). They have been classified into 11 genera (2); among them, 7 genera infecting mammals include *Norovirus*, *Sapovirus*, *Lagovirus*, *Vesivirus*, *Nebovirus* (1), *Recovirus* (3), and *Valovirus* (4); 2 genera infecting birds include *Bavovirus* (5, 6) and *Nacovirus* (6–8); and 2 genera infecting fish include *Salovirus* (9) and *Minovirus* (10). Sapoviruses and noroviruses are major human and animal pathogens that cause acute gastroenteritis (1, 11). Sapoviruses are currently classified into five genogroups (genogroups I to V) based on the complete sequence of the capsid; genogroups I, II, IV, and V are known to infect humans, and genogroup III infects porcine species (11–13). Porcine sapovirus (PSaV) Cowden strain is the only strain within the genus *Sapovirus* that replicates efficiently *in vitro* in a porcine kidney cell line supplemented with porcine intestinal contents or bile acids, specifically glycochenodeoxycholic acid (GCDCA) (14, 15). Thus, this strain is of particular interest in the study of sapovirus pathogenesis and molecular mechanisms at the level of virus-host interactions.

The gastrointestinal epithelium acts as a barrier between the luminal contents and the underlying tissue and is normally protected from pathogen invasion by the cell junctions. This is achieved through adhesion between neighboring cells, while allowing for the movement of ions and small molecules (16, 17). Tight junctions (TJs) are the most apical component of the apical junctional complex (AJC), which also includes adherent junctions and desmosomes (16, 17). TJs play a pivotal role in maintaining the barrier integrity of polarized epithelial cells, controlling the diffusion of molecules through the paracellular space, and acting as a physical barrier between the apical and basolateral domains (17, 18). TJ alterations have been shown to result in decreased transepithelial electrical resistance (TER) and increased paracellular permeability (17, 19), subsequently leading to various diseases such as inflammatory bowel disease, vasogenic edema, and certain cancers (18, 20–22). Many pathogens have evolved to manipulate the TJs and have hijacked buried junctional structures for their own purposes. They use the TJ proteins either as receptors for attachment and entry or as factors to facilitate virus entry (17, 18, 23, 24).

Regulation of TJ assembly and disassembly could be mediated by specific signaling pathways with signaling molecules such as RhoA, protein kinase C (PKC), protein kinase A (PKA), myosin light chain kinase (MLCK), mitogen-activated protein kinases (MAPKs), phosphatases, and phosphoinositide 3-kinase (18, 24–26). In particular, dissociation of TJs is induced primarily by contraction of the actomyosin ring, which is a dense circumferential belt formed by F-actin and myosin II at the level of the TJ and adherens junction (25, 27). Activation of the actomyosin ring is mediated directly via phosphorylation of a 20-kDa myosin II regulatory light chain (MLC) protein or indirectly via inhibition of MLC dephosphorylation through activation of the regulatory subunit of MLC phosphatase (MYPT). Finally, phosphorylation of MLC plays a central role in the activated RhoA/Rho-associated protein kinase (ROCK)/phosphorylated MLC (pMLC), PKC/MLCK/pMLC, and RhoA/ROCK/MYPT signaling pathways, thus providing the force for disruption of TJs (25, 27). Many viruses, such as rotavirus A (RVA), especially use the RhoA/ROCK/pMLC signaling pathway to dissociate TJs and then to enter the cells after binding to TJ molecules (28, 29).

Recently, we showed that TJs of polarized epithelial cells are impaired during the early stage of PSaV infection, as indicated by a decrease in TER and an increase in paracellular permeability (30). PSaV-induced TJ dissociation resulted in exposure of the TJ protein occludin, which binds to PSaV as a coreceptor (30). The PSaV-occludin complex is then internalized to the early and late endosomes in a small GTPase-, Rab5-, and Rab7-dependent manner (30). Claudin-1 facilitates the entry of PSaV into cells (30). However, the cellular signaling pathways involved in PSaV-induced TJ disruption are still undetermined. Here, we demonstrate that the PSaV Cowden strain induced phosphorylation of MLC protein via the RhoA/ROCK signaling pathway but independent of the PKC/MLCK and RhoA/ROCK/MYPT signaling pathways. Inhibition of the RhoA/ROCK/MLC signaling pathway in virus-infected polarized porcine kidney epithelial (LLC-PK) cells restored the decreased TER and increased paracellular permeability, intracellular translocation of occludin, and lateral membrane lipid diffusion.

## RESULTS

### PSaV activates pMLC via the RhoA/ROCK signaling pathway in polarized epithelial cells.

**FIG 1:**
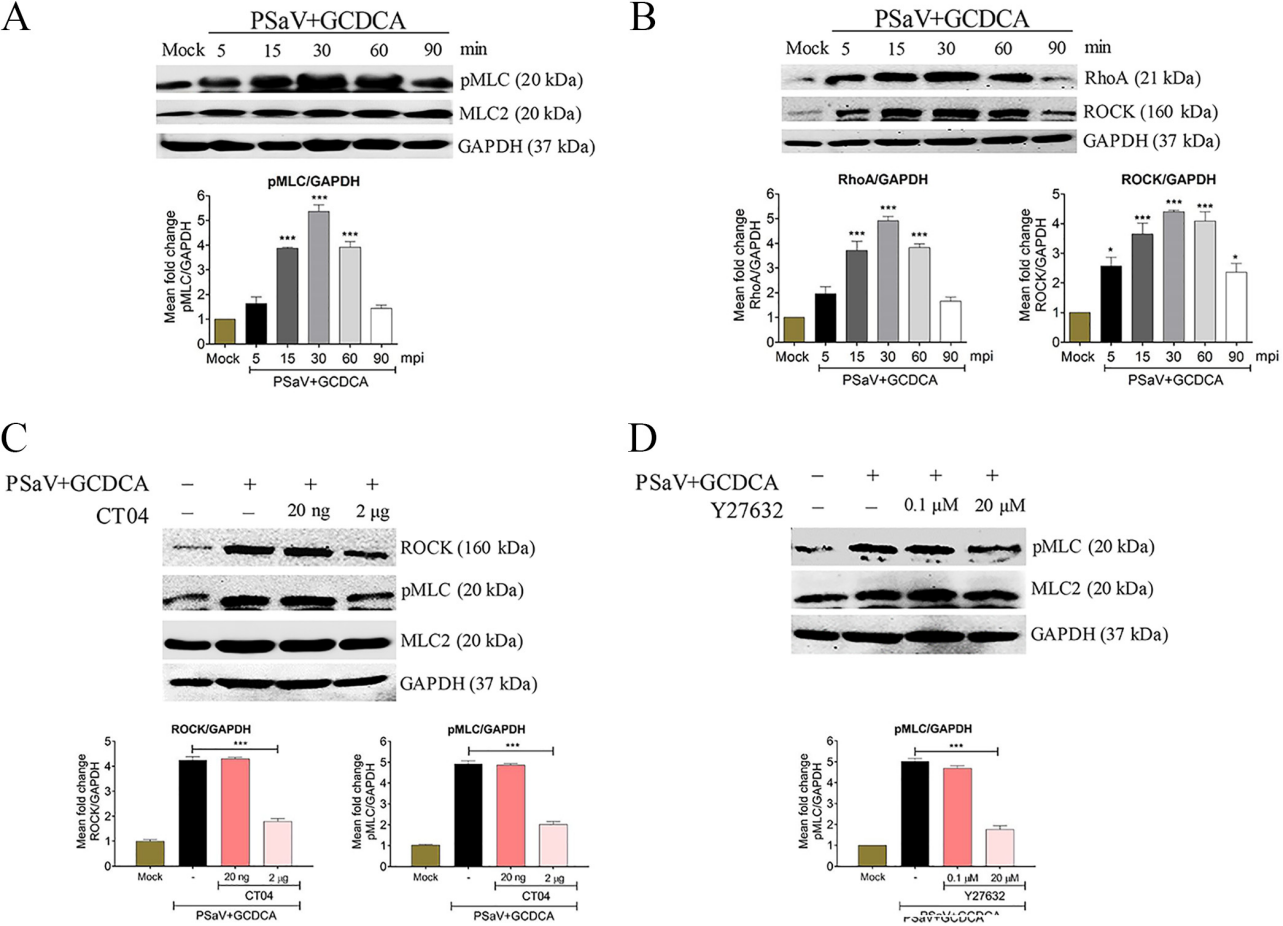
Early PSaV infection induces phosphorylation of MLC in a RhoA/ROCK-dependent manner in polarized LLC-PK cells. (A and B) Confluent LLC-PK cells were mock infected or infected with the PSaV Cowden strain (MOI of 1) in the presence of 200 μM GCDCA, and cells were harvested at the indicated time points. (A) Cell lysates were subjected to Western blotting to determine the expression levels of pMLC and MLC2 using the relevant antibodies or (B) were subjected to a RhoA activation assay to measure RhoA activation with an anti-RhoA antibody. The expression level of ROCK in the cell lysate was analyzed by Western blotting using an antibody against ROCK. (C and D) Confluent LLC-PK cells were mock treated or pretreated with RhoA inhibitor (CT04) (C) or ROCK inhibitor (Y27632) (D) at the indicated doses for 1 h at 37°C. These cells were then mock infected or infected with PSaV Cowden strain (MOI of 1) in the presence of 200 μM GCDCA. Cell lysates were harvested at 30 mpi, and the expression levels of ROCK and/or pMLC were evaluated by Western blotting. GAPDH was used as a loading control. Representative images of different gels from each group are presented. Data are presented as means ± standard errors of the mean from three independent experiments. The band intensities of pMLC, RhoA, and ROCK, relative to GAPDH, were determined by densitometric analysis across three independent experiments and statistically analyzed and are shown as graphs below each Western blot. Differences were evaluated using a one-way ANOVA. *, *P < *0.05; ***, *P < *0.0001.

We reported previously that early PSaV infection in LLC-PK cells markedly induced dissociation of TJs to expose its coreceptor occludin (30). pMLC induces actomyosin contraction, resulting in TJ disruption (25, 27). We first examined whether the early stage of PSaV infection induces the phosphorylation of MLC. Confluent LLC-PK cells were either mock infected or infected with PSaV Cowden strain at a multiplicity of infection (MOI) of 1 in the presence of 200 μM GCDCA. Western blot analysis with the harvested cell lysates was performed using a specific antibody against pMLC (Ser19). In contrast to mock-infected LLC-PK cells, in which MLC2 remained unchanged during the entire experiment, pMLC was activated in PSaV-infected cells as early as 15 min postinfection (mpi), peaked at 30 mpi, was sustained at a high level until 60 mpi, and declined thereafter (Fig. 1A).

**FIG 2:**
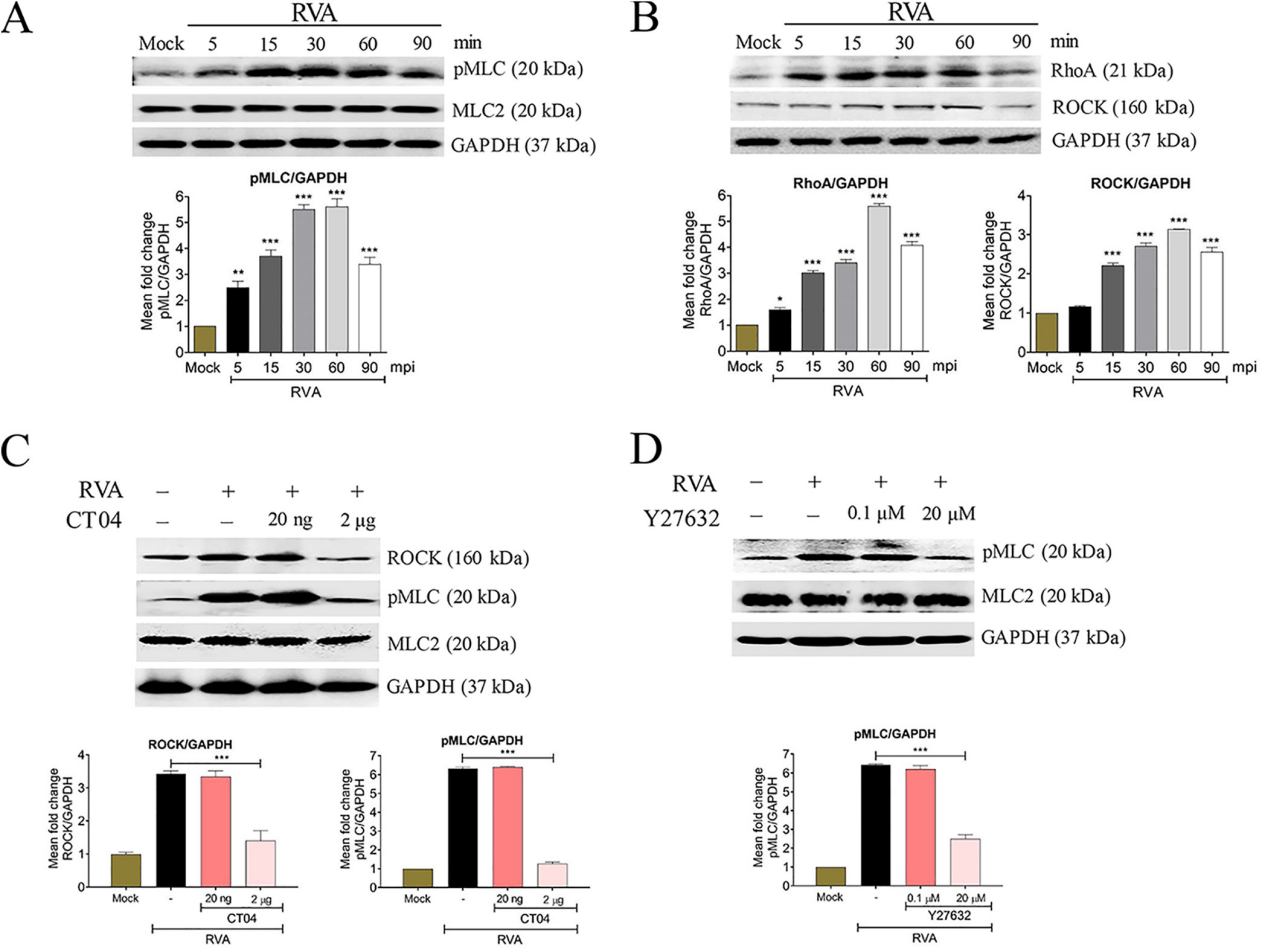
Early RVA infection induces phosphorylation of MLC in a RhoA/ROCK-dependent manner in polarized MDCK cells. (A and B) Confluent MDCK cells were mock infected or infected with bovine RVA NCDV strain (MOI of 10), and cells were harvested at the indicated time points. (A) Cell lysates were subjected to Western blotting to determine the expression levels of pMLC and MLC2 using the relevant antibodies or (B) were subjected to a RhoA activation assay to measure RhoA activation with an anti-RhoA antibody. The expression level of ROCK in the cell lysate was analyzed via Western blotting by using an antibody against ROCK. (C and D) Confluent MDCK cells were mock treated or pretreated with RhoA inhibitor (CT04) (C) or ROCK inhibitor (Y27632) (D) at the indicated doses for 1 h at 37°C. These cells were then mock infected or infected with bovine RVA NCDV strain (MOI of 10). Cell lysates were harvested 1 h postinfection, and the expression levels of ROCK and/or pMLC were evaluated via Western blotting. GAPDH was used as a loading control. Representative images of different gels from each group are presented. Data are presented as means ± standard errors of the mean from three independent experiments. The band intensities of pMLC, RhoA, and ROCK, relative to GAPDH, were determined by densitometric analysis across three independent experiments and statistically analyzed and are shown as graphs below each Western blot. Differences were evaluated using a one-way ANOVA. *, *P < *0.05; **, *P < *0.001; ***, *P < *0.0001.

To further identify the upstream effectors of pMLC in PSaV-infected cells, confluent LLC-PK cells were infected at the indicated time points as stated above. The cell lysates were used in a RhoA activation assay using Rhotekin RBD agarose beads, and RhoA was then detected by Western blotting using a specific antibody against RhoA. The expression levels of ROCK, a molecule downstream of RhoA, were analyzed in the cell lysate by Western blotting using its specific antibody (31–34). Our results indicated that PSaV-induced early activation of RhoA and ROCK in confluent LLC-PK cells was similar to PSaV-induced early activation of pMLC (Fig. 1B). As a positive control, we used bovine RVA strain NCDV, which induces the phosphorylation of MLC in a RhoA/ROCK-dependent manner (29). As expected, RVA induced the activation of pMLC (Fig. 2A), RhoA, and ROCK (Fig. 2B) signaling molecules at early times of infection, peaking at 60 mpi.

**FIG 3:**
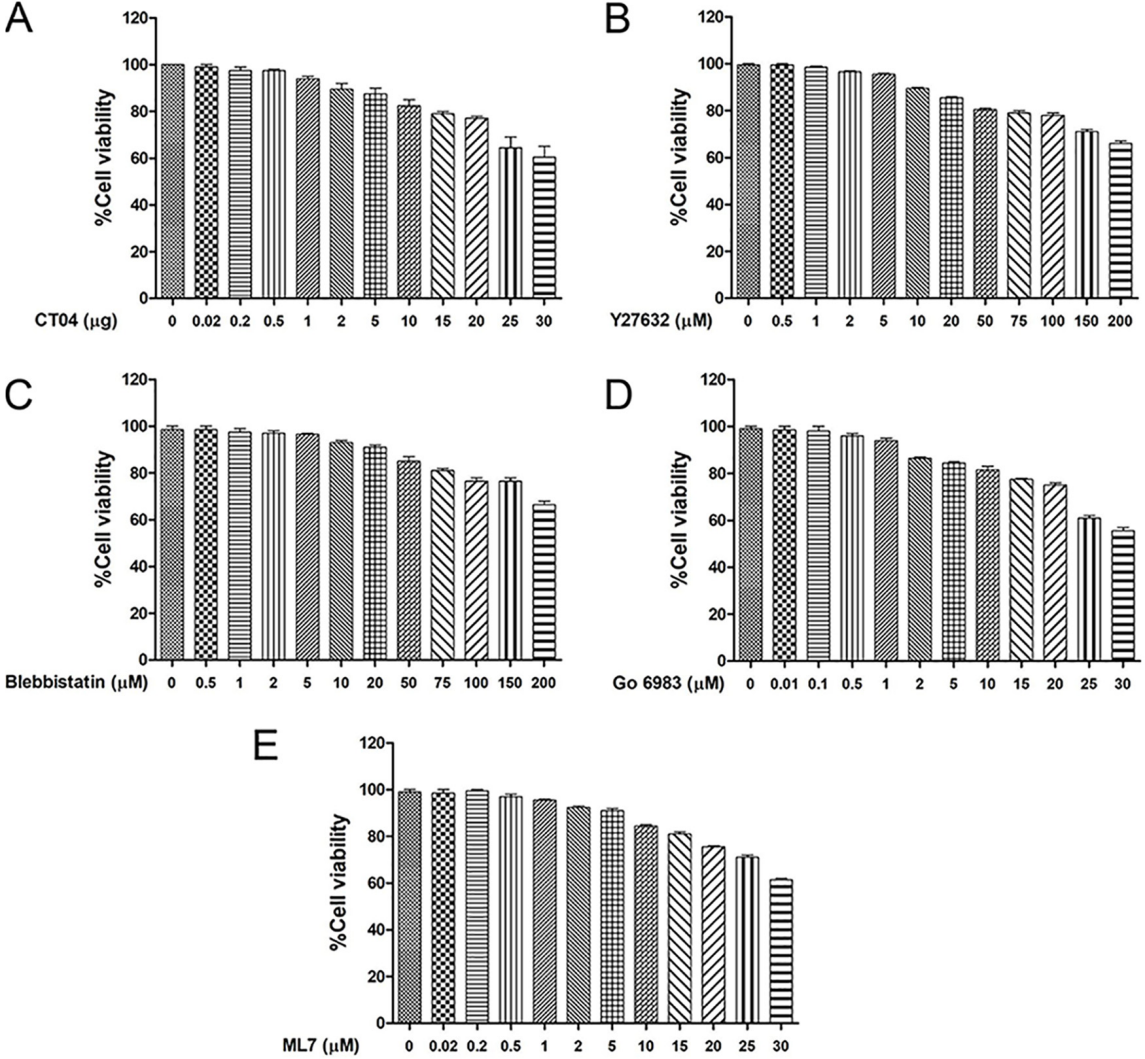
Determination of the cytotoxicity of chemicals by the MTT assay. (A to E) LLC-PK cells grown in 96-well plates were incubated with various concentrations of the indicated chemicals in triplicate for 24 h at 37°C. Afterward, the chemical-containing media were thoroughly removed and replaced with 200 μl of MTT solution for 4 h at 37°C. Each well was incubated with 150 μl of DMSO for 10 min at 20°C. Cell viability was measured as OD_570_ using an ELISA reader.

To confirm the results described above, LLC-PK cells were pretreated for 1 h with inhibitors specific to RhoA (CT04) or ROCK (Y27632) (Fig. 1C and D), incubated with PSaV for 30 min, and analyzed for the activation of ROCK and/or pMLC by Western blotting. The concentration of each inhibitor used was noncytotoxic and did not affect the viability of LLC-PK cells, as assessed through the 3-(4,5-dimethylthiazol-2-yl)-2,5-diphenyltetrazolium bromide (MTT) assay (Fig. 3). The expression level of ROCK was decreased 2-fold following treatment with the RhoA inhibitor CT04, and that of pMLC was decreased 3-fold after treatment with CT04 at 2 μg or Y27632 at 20 μM (Fig. 1C and D). Similarly, the expression of RVA-induced ROCK or pMLC was reduced after treatment with CT04 at 2 μg or Y27632 at 20 μM (Fig. 2C and D). Taken together, these data suggested that pMLC was activated in PSaV-infected cells in a RhoA/ROCK-dependent manner.

### PSaV triggers activation of the RhoA/ROCK/pMLC signaling pathway in the absence of GCDCA.

**FIG 4:**
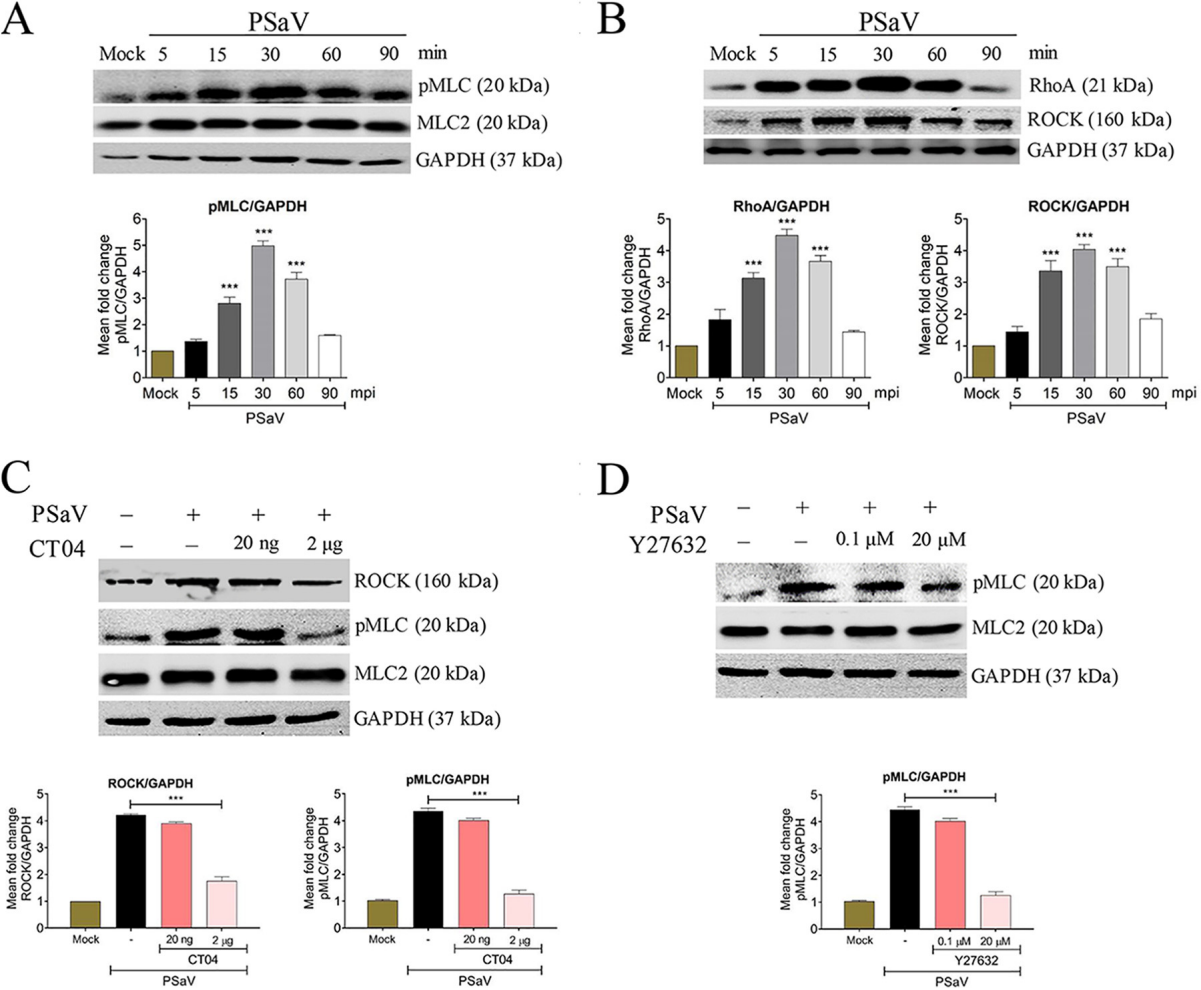
PSaV infection activates the RhoA/ROCK/MLC signaling pathway in the absence of GCDCA. (A and B) PSaV Cowden strain (MOI of 1), in the absence of GCDCA, was inoculated into confluent LLC-PK cells, and cells were harvested at the indicated time points. (A) Cell lysates were subjected to Western blotting to determine the expression levels of pMLC and MLC2 using the relevant antibodies or (B) were subjected to a RhoA activation assay to measure RhoA activation with an anti-RhoA antibody. The expression level of ROCK in the cell lysate was analyzed by Western blotting using an antibody against ROCK. (C and D) Confluent LLC-PK cells were pretreated with or without RhoA inhibitor (CT04) (C) or ROCK inhibitor (Y27632) (D) at the indicated doses for 1 h at 37°C and then mock infected or infected with PSaV Cowden strain (MOI of 1) in the absence of GCDCA. Cell lysates were harvested at 30 mpi, and the expression levels of ROCK and/or pMLC were evaluated via Western blotting. GAPDH was used as a loading control. Representative images of different gels from each group are presented. Data are presented as means ± standard errors from three independent experiments. The band intensities of pMLC, RhoA, and ROCK, relative to GAPDH, were determined using densitometric analysis across three independent experiments and statistically analyzed and are shown as graphs below each Western blot. Differences were evaluated using a one-way ANOVA. ***, *P < *0.0001.

PSaV alone without the addition of GCDCA could disrupt epithelial TJs (30). Therefore, we assessed whether PSaV infection in the absence of GCDCA promotes the activation of RhoA/ROCK/MLC signaling. LLC-PK cells infected with PSaV in the absence of GCDCA for the indicated times showed activation of pMLC, RhoA, and ROCK as early as 15 mpi, peaking at 30 mpi (Fig. 4A and B). In addition, pretreatment of cells with the RhoA inhibitor CT04 at 2 μg reduced the expression levels of ROCK and pMLC 2- and 3-fold, respectively, while pretreatment with the ROCK inhibitor Y27632 at 20 μM reduced the expression of pMLC 3-fold in a dose-dependent manner (Fig. 4C and D).

### PSaV-induced early activation of pMLC is independent of the RhoA/ROCK/MYPT and PKC/MLCK signaling pathways.

**FIG 5:**
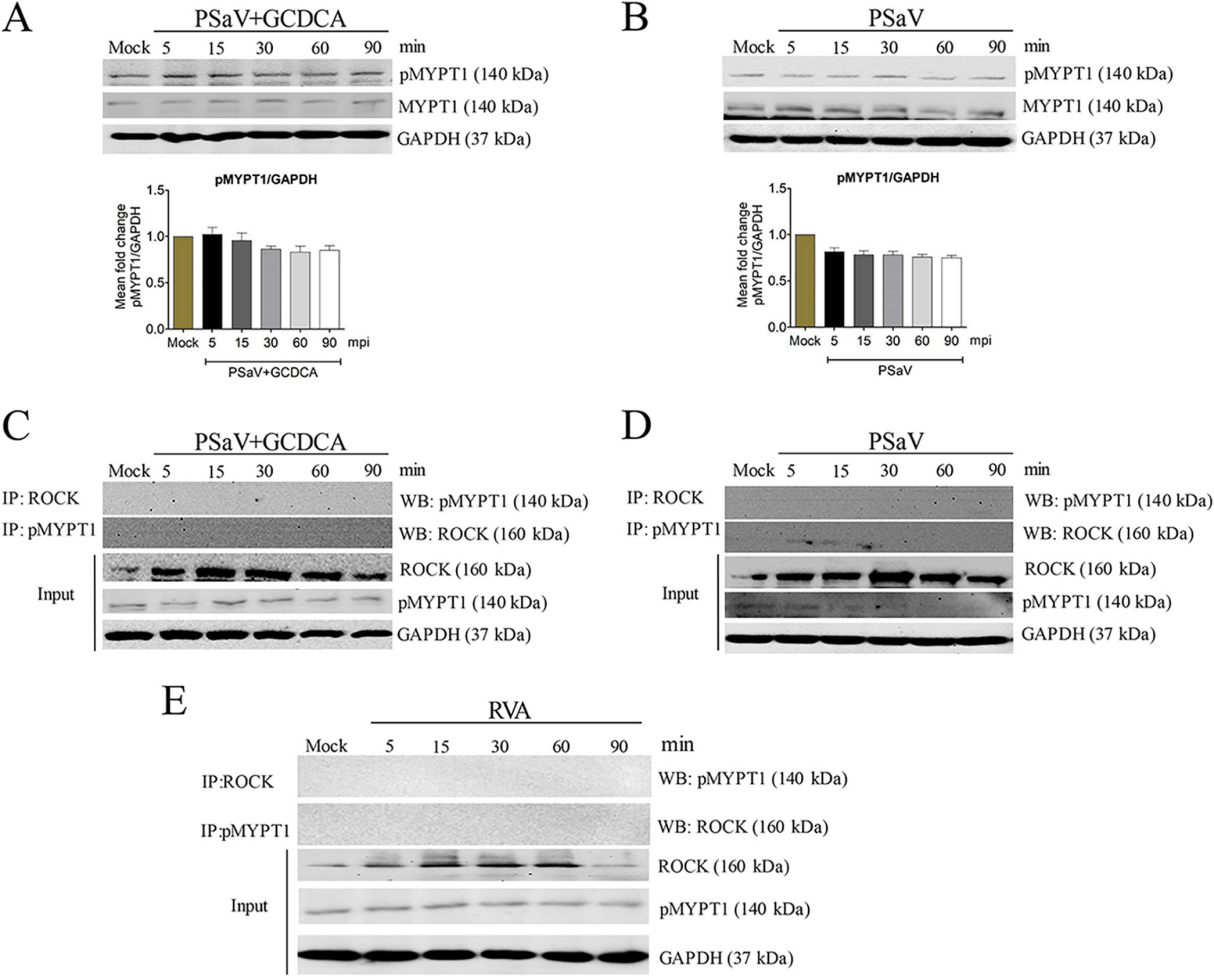
MYPT subunit is not activated in LLC-PK cells during the early stage of PSaV infection. (A and B) Confluent LLC-PK cells were mock infected or infected with PSaV Cowden strain (MOI of 1) in the presence (A) or absence (B) of GCDCA. Cells were then harvested at the indicated time points. The cell lysates were subjected to Western blotting to determine the expression levels of pMYPT and MYPT using the relevant antibodies. GAPDH was used as a loading control. Representative images of different gels from each group are presented. Data are presented as means ± standard errors from three independent experiments. The band intensities of pMYPT1, relative to GAPDH, were determined using densitometric analysis across three independent experiments and statistically analyzed and are shown as graphs below each Western blot. Differences were evaluated using a one-way ANOVA. (C to E) Lysates of LLC-PK cells either mock infected or infected with PSaV Cowden strain (MOI of 1) in the presence (C) or absence (D) of GCDCA at the indicated time points and lysates of MDCK cells either mock infected or infected with bovine RVA NCDV strain (MOI of 10) (E) were immunoprecipitated using antibodies specific for ROCK or pMYPT. Coimmunoprecipitated proteins were analyzed via Western blotting to detect pMYPT or ROCK by using the relevant antibodies. Representative images of different gels from three independent experiments are presented. IP, immunoprecipitation; WB, Western blotting.

ROCK can induce phosphorylation of MYPT at its Thr696 and Thr853 residues, resulting in suppression of myosin phosphatase and hence the increase of MLC phosphorylation (25, 27). To examine whether early infection of PSaV induces the phosphorylation of MYPT, confluent LLC-PK cells were infected with PSaV in the presence or absence of GCDCA at the indicated time points as stated above, and the cell lysates were used for the detection of phosphorylated MYPT (pMYPT) via Western blotting. We did not observe any change in the activation levels of pMYPT in PSaV-infected cells throughout the tested time points, either in the presence or in the absence of GCDCA (Fig. 5A and B). To corroborate the results described above, confluent LLC-PK cells were either mock infected or infected with PSaV, and cell lysates were immunoprecipitated with antibodies specific for pMYPT or ROCK at the indicated times. Consistently, both antibodies did not precipitate their counterparts (Fig. 5C and D), as also observed in RVA strain NCDV-infected Madin—Darby canine kidney (MDCK) cells (Fig. 5E). These data suggested that PSaV-induced early activation of pMLC was independent of the RhoA/ROCK/pMYPT pathway.

**FIG 6:**
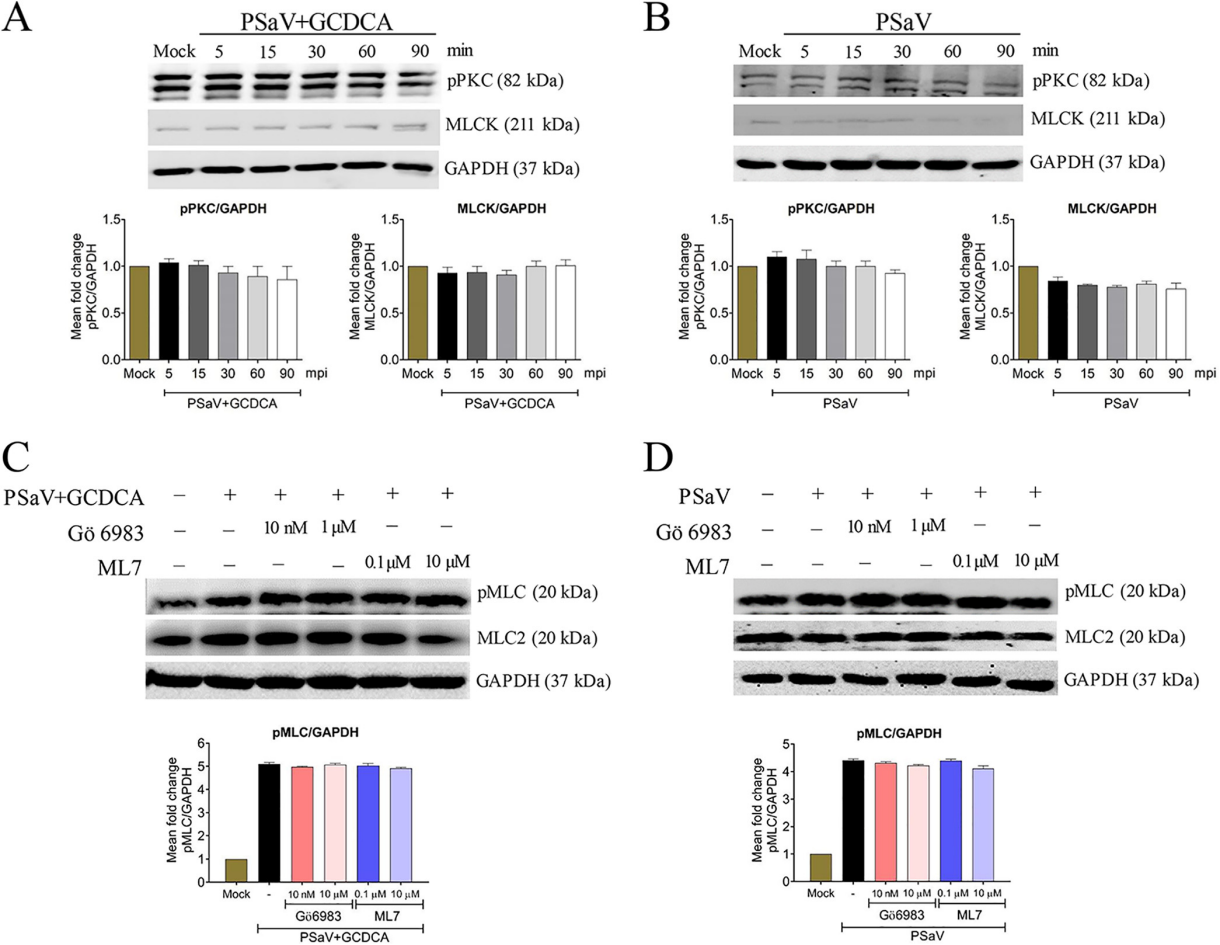
PSaV-induced activation of pMLC is independent of the PKC/MLCK signaling pathway. (A and B) Confluent LLC-PK cells were either mock infected or infected with PSaV Cowden strain (MOI of 1) in the presence (A) or absence (B) of GCDCA for the indicated time points. Cell lysates were subjected to Western blotting to detect pPKC and MLCK using the relevant antibodies. GAPDH was used as a loading control. (C and D) Confluent LLC-PK cells were either mock treated or pretreated with the PKC inhibitor Gö 6983 or the MLCK inhibitor ML7 for 1 h at 37°C and then infected with PSaV Cowden strain (MOI of 1) in the presence (C) or absence (D) of GCDCA. Cell lysates were harvested after 30 min, and the expression level of pMLC was evaluated via Western blotting. GAPDH was used as a loading control. Representative images of different gels from each group are presented. Data are presented as means ± standard errors from three independent experiments. The band intensities of pPKC and MLCK, relative to GAPDH, were determined using densitometric analysis across three independent experiments and statistically analyzed and are shown as graphs below each Western blot. Differences were evaluated using a one-way ANOVA.

**FIG 7:**
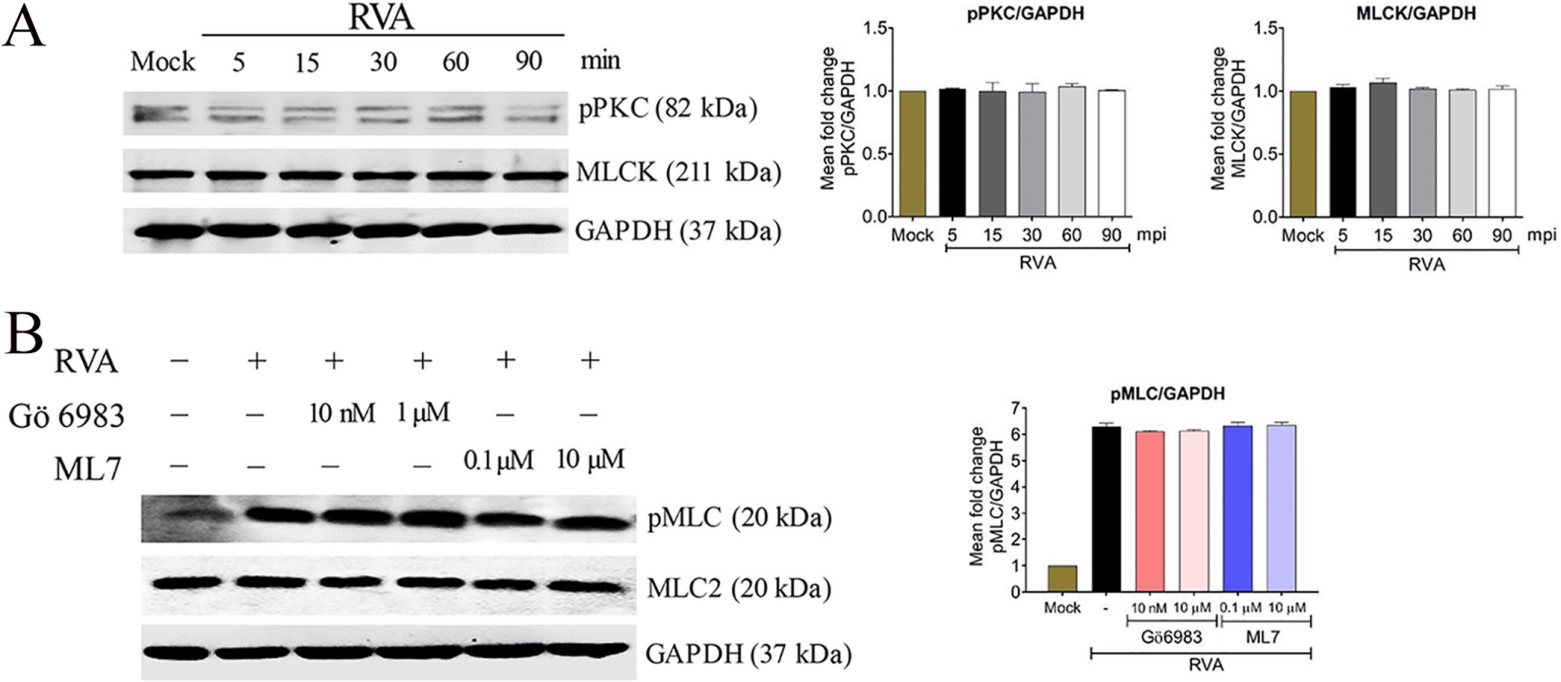
Rotavirus-induced activation of pMLC is independent of the PKC/MLCK signaling pathway. (A) Confluent MDCK cells were either mock infected or infected with bovine RVA NCDV strain (MOI of 10) for the indicated time points. Cell lysates were subjected to Western blotting to detect pPKC and MLCK by using the relevant antibodies. GAPDH was used as a loading control. (B) Confluent MDCK cells were either mock treated or pretreated with the PKC inhibitor Gö 6983 or the MLCK inhibitor ML7 for 1 h at 37°C and then infected with bovine RVA NCDV strain (MOI of 10). Cell lysates were harvested after 1 h, and the expression level of pMLC was evaluated via Western blotting. GAPDH was used as a loading control. Representative images of different gels from three independent experiments are presented. Data are presented as means ± standard errors from three independent experiments. The band intensities of pPKC and MLCK, relative to GAPDH, were determined using densitometric analysis across three independent experiments and statistically analyzed and are shown as graphs below each Western blot. Differences were evaluated using a one-way ANOVA.

It has long been recognized that the PKC/MLCK pathway affects the epithelial and endothelial barriers through MLC activation (25). Therefore, we next checked whether the phosphorylation of MLC was mediated through upregulation of the upstream PKC/MLCK signaling cascade (35–37). For this, each mock- or PSaV-infected cell lysate was subjected to Western blotting with antibodies specific for phosphorylated PKC (pPKC) and MLCK. The levels of pPKC and MLCK did not change in virus-infected cells in the presence or absence of GCDCA, relative to those in mock-infected cells (Fig. 6A and B). In addition, pretreatment of confluent LLC-PK cells with the PKC inhibitor Gö 6983 or the MLCK inhibitor ML7 did not alter the level of virus-activated pMLC (Fig. 6C and D). Similarly, the expression levels of pPKC and MLCK were unchanged in RVA-infected cells (Fig. 7A), and the RVA-activated pMLC levels did not change upon treatment with Gö 6983 or ML7 (Fig. 7B). These results suggested that PSaV activated pMLC independent of the PKC/MLCK signaling pathway.

### Inhibition of the RhoA/ROCK/MLC signaling pathway reduced PSaV infectivity and viral protein expression.

**FIG 8:**
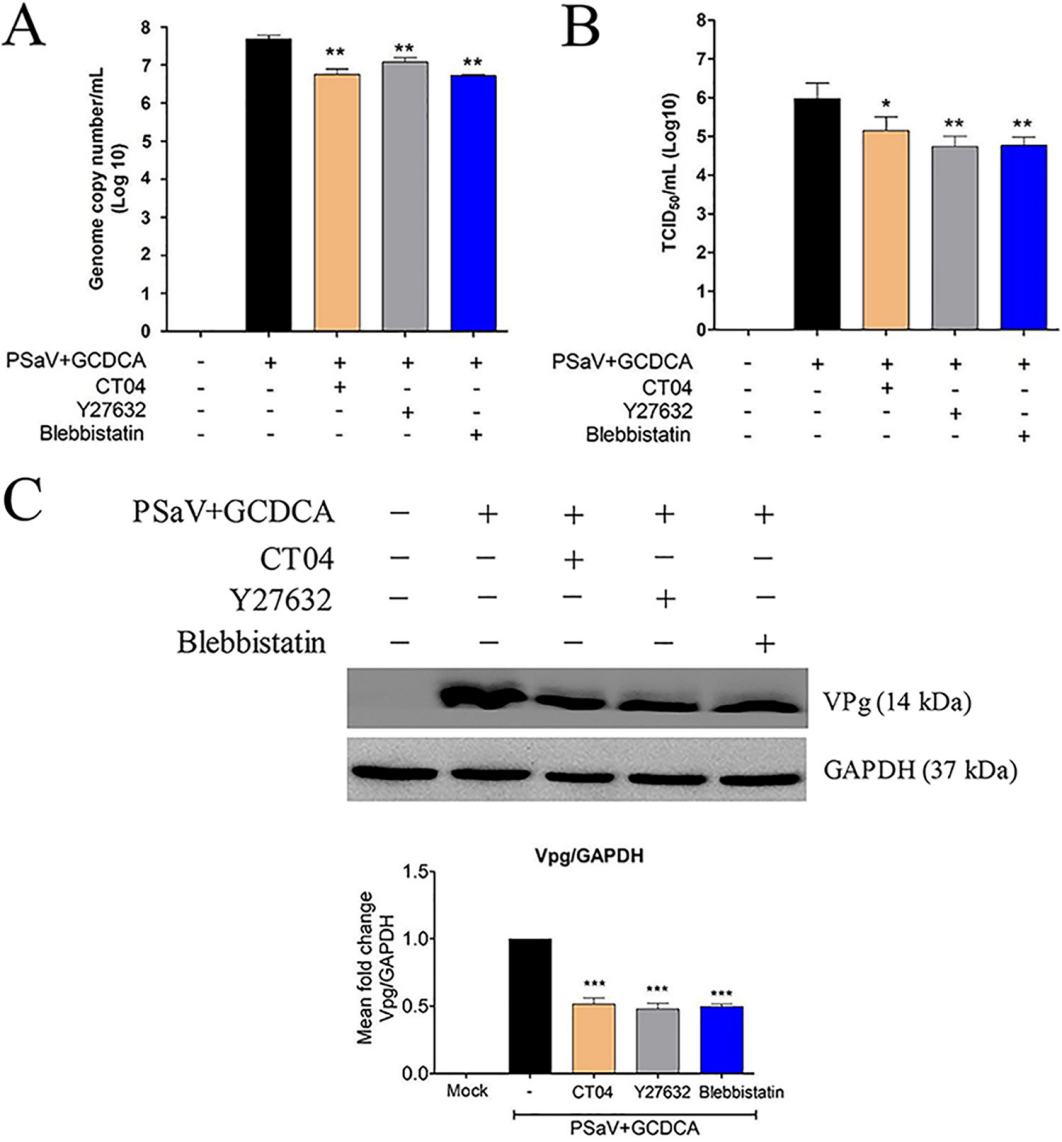
Inhibition of the RhoA/ROCK/MLC signaling pathway affects PSaV infectivity and viral protein expression. LLC-PK cells were mock treated or pretreated with noncytotoxic concentrations of RhoA inhibitor CT04 (2 μg), ROCK inhibitor Y27632 (20 μM), or MLC inhibitor blebbistatin (10 μM) for 1 h at 37°C and then infected with PSaV Cowden strain (MOI of 1) in the presence of 200 μM GCDCA for 36 h. (A) Genome copy number was determined via RT-qPCR. (B) Virus titers were determined using TCID_50_ using cell lysates produced by three cycles of freezing and thawing. (C) VPg was detected via Western blotting. PSaV genome copy numbers, viral titers, and VPg expression levels were described in Materials and Methods. GAPDH was used as a loading control. The band intensity of VPg, relative to GAPDH, was determined using densitometric analysis across three independent experiments and statistically analyzed and is shown as a graph below the Western blot. All experiments, including RT-qPCR and TCID_50_ assays, were also performed in triplicate. Representative images of different gels from each group are presented. Data are presented as means ± standard errors from three independent experiments. Differences were evaluated using a one-way ANOVA. *, *P < *0.05; **, *P < *0.001; ***, *P < *0.0001.

Activation of the RhoA/ROCK/MLC signaling pathway during the early stage of PSaV infection could be involved in the PSaV life cycle, and blocking this pathway could eventually affect virus replication. Therefore, we tested whether these signaling molecules are involved in the PSaV life cycle. We pretreated LLC-PK cells for 1 h with specific inhibitors against RhoA (CT04), ROCK (Y27632), or MLC (blebbistatin), infected them with PSaV Cowden strain for 36 h, and checked the infectivity of the viral progeny. Pretreatment with CT04, Y27632, or blebbistatin showed an ∼0.8- to 1-log-unit reduction in the genome copy number (Fig. 8A), resulting in an ∼0.8- to 1.2-log-unit decline in the infectivity of viral progeny (Fig. 8B) and an ∼50% reduction in viral protein expression (Fig. 8C). These data suggest that PSaV-induced early activation of the RhoA/ROCK/MLC signaling pathway eventually affects PSaV infectivity and progeny production.

### PSaV-induced alteration in TER in polarized epithelial cells is dependent on the RhoA/ROCK/MLC pathway.

**FIG 9:**
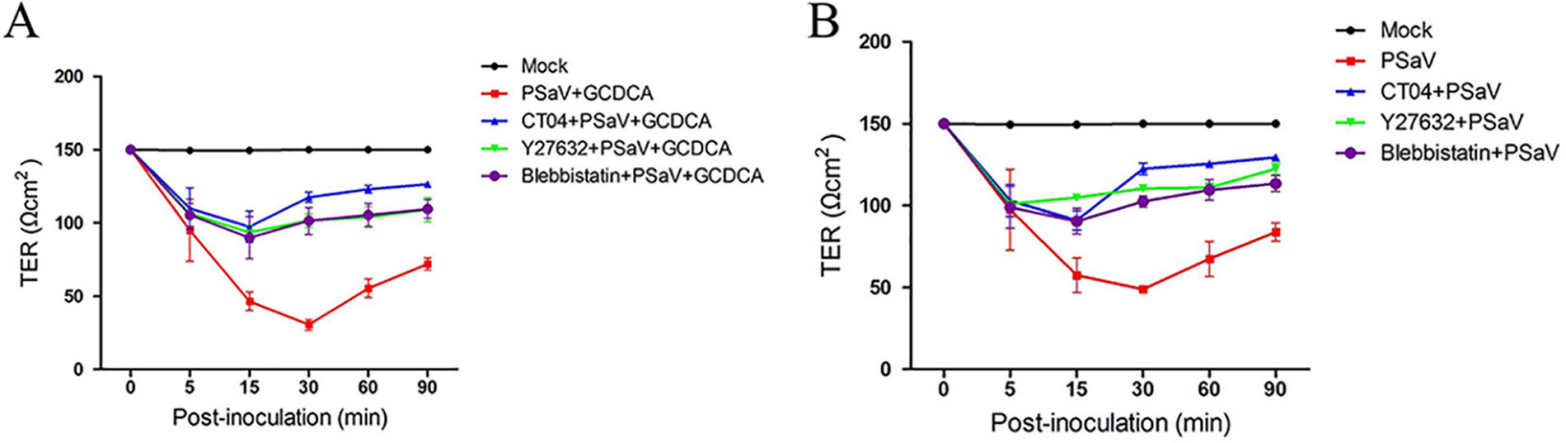
Inhibition of the RhoA/ROCK/MLC pathway restores the TER of the TJ in PSaV-infected polarized epithelial cells. (A and B) Confluent LLC-PK cells grown on Transwell filters were pretreated with or without RhoA inhibitor CT04 (2 μg), ROCK inhibitor Y27632 (20 μM), or MLC inhibitor blebbistatin (10 μM) for 1 h at 37°C and then mock inoculated or inoculated with PSaV Cowden strain (MOI of 50) in the presence (A) or absence (B) of GCDCA, followed by determination of TER at the indicated time points. Net TER was calculated by subtracting the background (membrane filter without cells) and multiplying the resistance (ohms) by the area (0.33 cm^2^) of the filter. All experiments were performed in triplicate. Data are presented as means ± standard errors from three independent experiments.

We previously showed that PSaV, with or without GCDCA supplementation, is capable of decreasing TER in polarized epithelial monolayers through the breakdown of TJs (30). Therefore, we examined whether the RhoA/ROCK/MLC signaling cascade is responsible for the reduction of TER associated with PSaV infection. To assess this, confluent polarized LLC-PK cells grown on a Transwell polyester (polyethylene terephthalate [PET]) membrane insert were either mock treated or pretreated for 1 h with RhoA inhibitor (CT04), ROCK inhibitor (Y27632), or MLC inhibitor (blebbistatin). The cells were then infected with PSaV or not in the presence or absence of GCDCA, and the TER was measured at the indicated time points. As expected, the TER across the mock-infected cells remained unchanged (Fig. 9A and B). However, infection with PSaV Cowden strain, regardless of the presence of GCDCA, resulted in a significant decrease in the TER in LLC-PK monolayers at 30 mpi (Fig. 9A and B), consistent with our previous results (30). Inhibition of RhoA, ROCK, or MLC restored the decrease in TER in LLC-PK monolayers infected with PSaV by 3.3- to 3.9-fold in the presence of GCDCA or by 2.1- to 2.5-fold in the absence of GCDCA (Fig. 9A and B), suggesting that the RhoA/ROCK/MLC pathway mediates the disruption of TJ function induced by the early stage of PSaV infection.

### PSaV-induced early activation of the RhoA/ROCK/MLC pathway modifies the distribution of TJ proteins.

**FIG 10:**
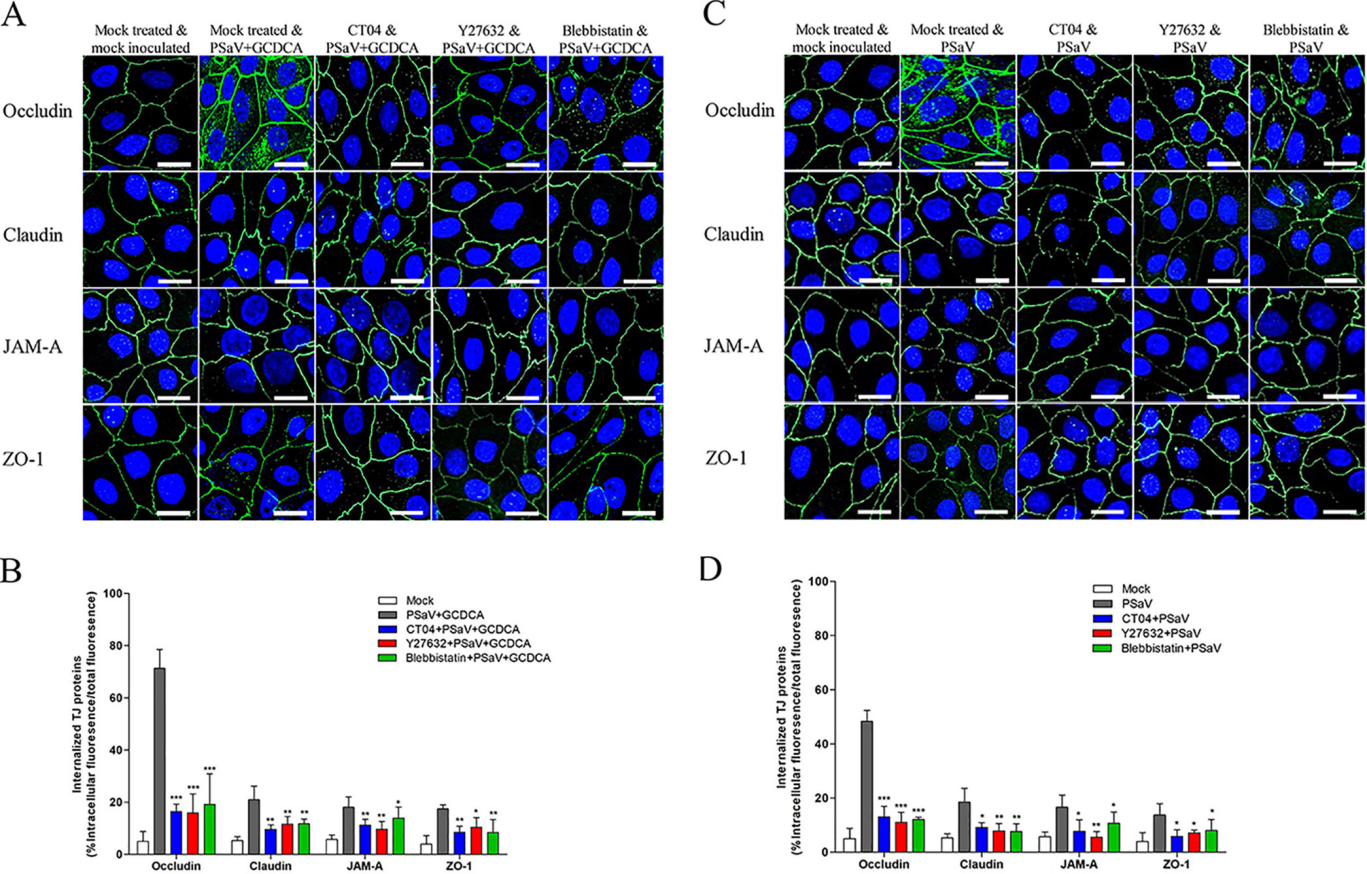
Distribution of occludin is altered by the PSaV-induced RhoA/ROCK/MLC signaling pathway. (A to D) Confluent LLC-PK cells were untreated or treated with RhoA inhibitor CT04 (2 μg), ROCK inhibitor Y27632 (20 μM), or MLC inhibitor blebbistatin (10 μM) for 1 h at 37°C and then mock inoculated or inoculated with PSaV Cowden strain (MOI of 50) in the presence (A and B) or absence (C and D) of GCDCA for 30 min. Cells were then fixed, permeabilized, and prepared for confocal microscopy using rabbit antioccludin, anticlaudin, anti-JAM-A, and anti-ZO-1 antibodies and relevant secondary antibodies. All experiments were performed in triplicate and representative images are shown. The scale bars correspond to 10 μm. (B and D) The internalized TJ molecules, shown as a percentage of intracellular fluorescence/total fluorescence, are presented as means ± standard errors from three independent experiments. Differences were evaluated using a one-way ANOVA. *, *P < *0.05; **, *P < *0.001; ***, *P < *0.0001.

**FIG 11:**
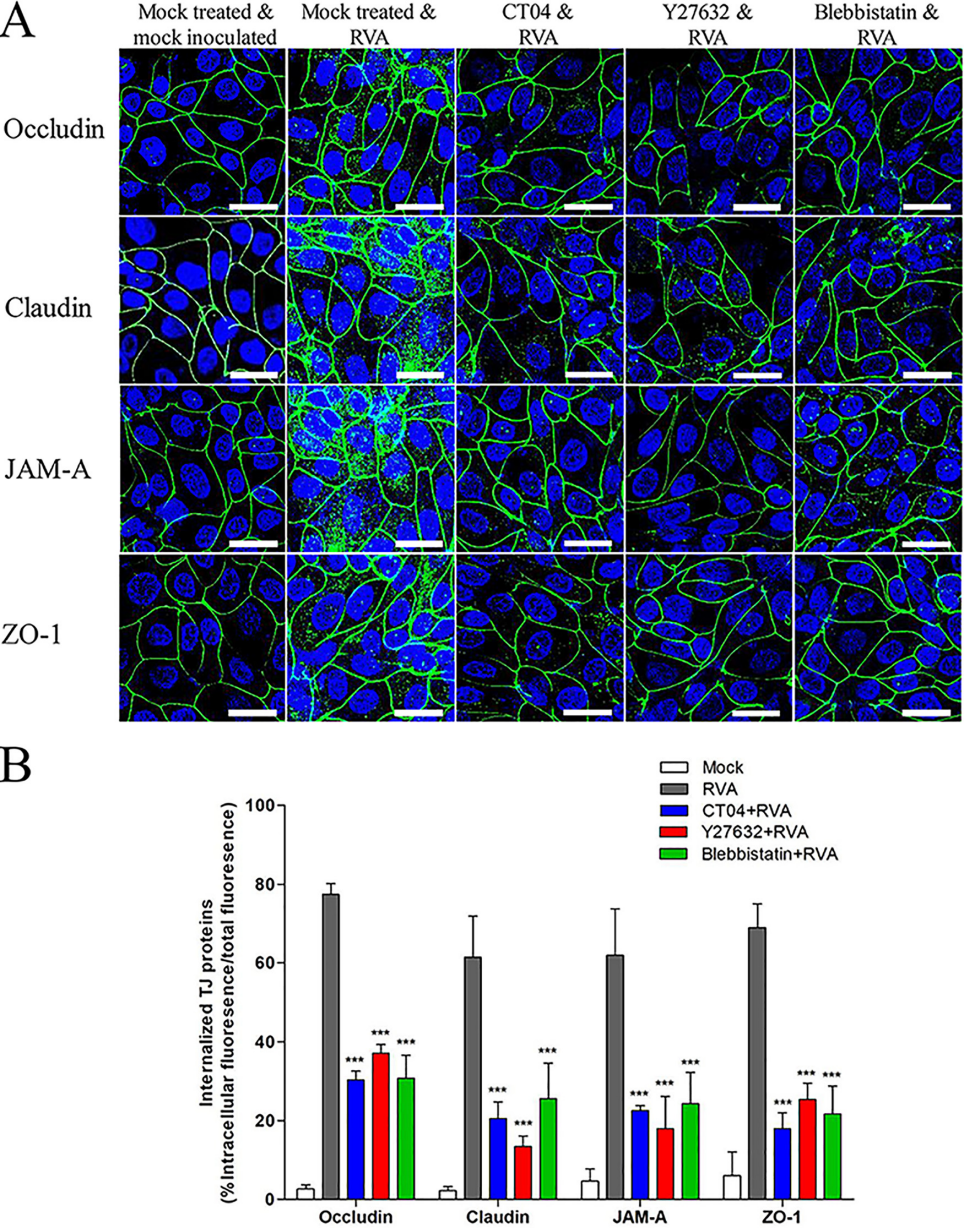
Distribution of the TJ proteins is altered by the rotavirus-induced RhoA/ROCK/MLC signaling pathway. (A and B) Confluent MDCK cells were untreated or treated with RhoA inhibitor CT04 (2 μg), ROCK inhibitor Y27632 (20 μM), or MLC inhibitor blebbistatin (10 μM) for 1 h at 37°C and then mock inoculated or inoculated with bovine RVA NCDV strain (MOI of 10) for 1 h. Cells were then fixed, permeabilized, and prepared for confocal microscopy using rabbit antioccludin, anticlaudin, anti-JAM-A, and anti-ZO-1antibodies and relevant secondary antibodies. All experiments were performed in triplicate, and representative images are shown. The scale bars correspond to 20 μm. (B) The internalized TJ molecules, shown as a percentage of intracellular fluorescence/total fluorescence, are presented as means ± standard errors from three independent experiments. Differences were evaluated using a one-way ANOVA. ***, *P < *0.0001.

We previously reported that PSaV-disrupted TJs in LLC-PK cells are associated with the relocalization of TJ proteins from the cell surface to the cytoplasm (30). In epithelial cells, the apical actomyosin ring associates with the AJC and controls the assembly and barrier properties of this complex (38, 39). Since many studies have linked phosphorylation of MLC to increased actin polymerization and stress fiber assembly and *in vivo* and *in vitro* reorganization of TJ proteins (40–42), we questioned whether the internalization of TJ proteins in PSaV-infected cells could be inhibited by blocking the RhoA/ROCK/MLC pathway. To verify this, LLC-PK cells were either mock treated or treated with RhoA inhibitor (CT04), ROCK inhibitor (Y27632), or MLC inhibitor (blebbistatin) and then mock infected or infected with PSaV in the presence or absence of GCDCA. In mock-treated and mock-infected cells, there was no change in the staining pattern of TJ proteins on the cell surface (Fig. 10). In contrast, LLC-PK monolayers infected with PSaV showed considerable and prominent rearrangement of occludin in the cytoplasm, compared with other TJ proteins, such as claudin, JAM-A, and ZO-1; it was more prominent in the presence (Fig. 10A) than in the absence (Fig. 10C) of GCDCA. In addition, infected cells supplemented with GCDCA (Fig. 10B) showed a higher percentage of internalized occludin in the cytoplasm than of claudin, JAM-A, and ZO-1, compared with the absence of GCDCA (Fig. 10D). These results are consistent with our previous results confirming that PSaV infection of polarized LLC-PK cells alters the distribution of occludin (30). However, pretreatment of LLC-PK cells with the aforementioned inhibitors partially reduced the translocation of occludin from the junctional area to the cytoplasm and consequently reduced the percentage of internalized occludin (Fig. 10). However, the TJ proteins (occludin, claudin, JAM-A, and ZO-1) were intracellularly distributed in RVA-infected MDCK cells and were reduced following treatment with RhoA inhibitor (CT04), ROCK inhibitor (Y27632), or MLC inhibitor (blebbistatin) (Fig. 11A and B). These data indicated that inhibition of the RhoA/ROCK/MLC signaling pathway partially reduced the intracellular translocation of occludin from the pericellular plasma junction to the cytosol.

### Inhibition of the RhoA/ROCK/MLC pathway restores the gate and fence functions of TJs disrupted by PSaV infection.

**FIG 12:**
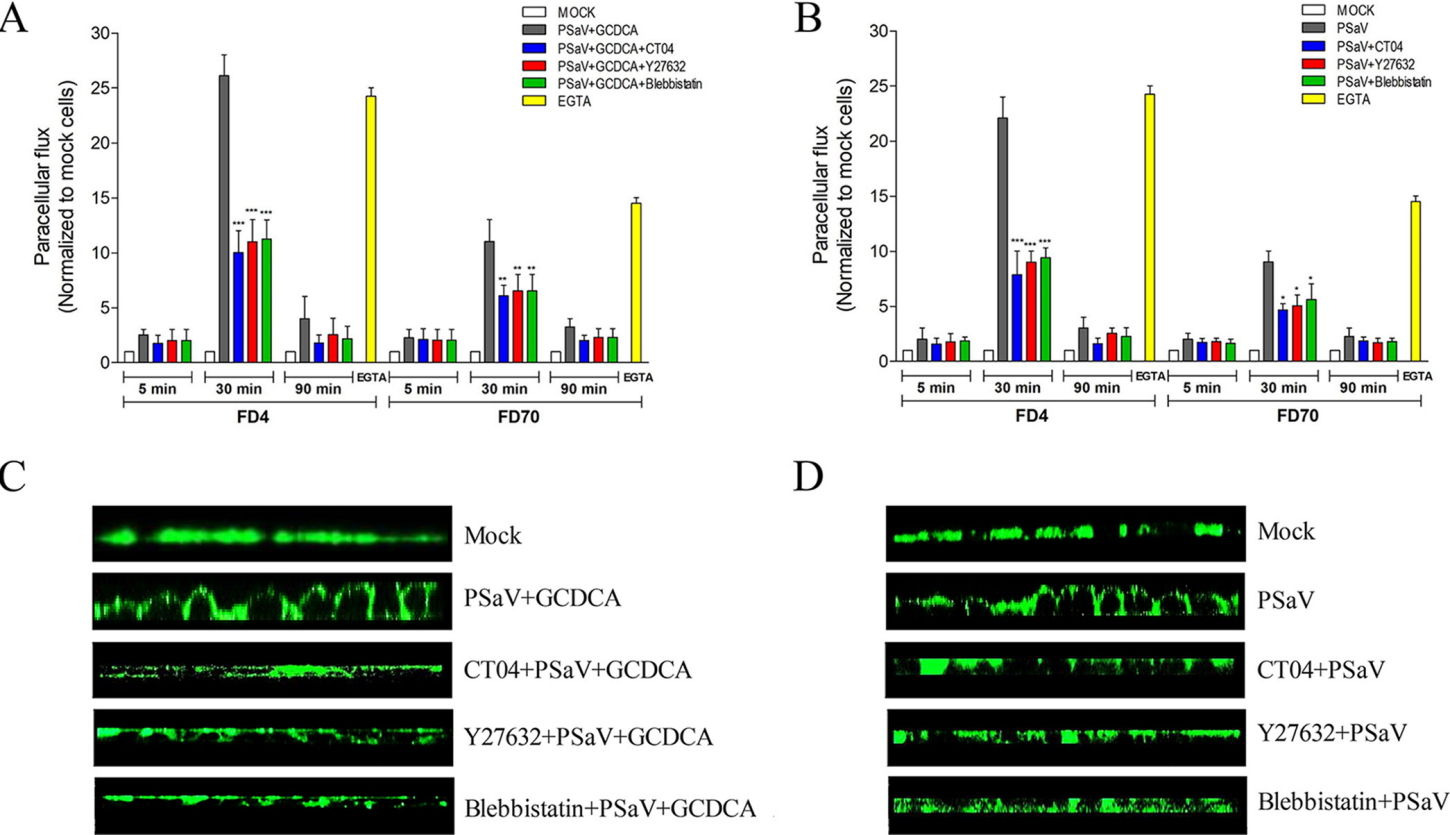
Inhibition of the RhoA/ROCK/MLC pathway restores the gate and fence functions of TJs in PSaV-infected polarized epithelial cells. Confluent LLC-PK cells grown on Transwell filters were untreated or treated with RhoA inhibitor CT04 (2 μg), ROCK inhibitor Y27632 (20 μM), or MLC inhibitor blebbistatin (10 μM) for 1 h at 37°C and then mock inoculated or inoculated with PSaV Cowden strain (MOI of 50) in the presence (A) or absence (B) of GCDCA for 5, 30, or 90 min. (A and B) Paracellular flux of FD4 and FD70 was measured in the apical-to-basolateral direction. The amount of FITC-dextran diffused to the basolateral side of the monolayer was normalized to the average obtained from control LLC-PK cells. As a positive control, confluent LLC-PK cells were treated for 10 min with 1.8 mM EGTA. All experiments were performed in triplicate. Data are presented as means ± standard errors from three independent experiments. Differences were evaluated using a one-way ANOVA. *, *P < *0.05; **, *P < *0.001; ***, *P < *0.0001. (C and D) The distribution of the membrane fluorescent marker BodipyFL-C_12_-sphingomyelin-BSA (5 nmol/ml) loaded onto the apical surface of LLC-PK cells was determined via z-sectioning using a confocal microscope. All experiments were performed in triplicate; panels C and D show representative results.

To verify the role of the PSaV-induced RhoA/ROCK/MLC signaling pathway in the integrity of TJs in LLC-PK cells, we next examined the change in the gate function of TJs by analyzing the paracellular permeability of nonionic tracers, 4-kDa and 70-kDa fluorescein isothiocyanate (FITC)-dextrans (FD4 and FD70, respectively), in LLC-PK cells. Consistent with the results of our previous study (30), the permeability of polarized LLC-PK cells to FD4 and FD70 was increased 26- and 11-fold, respectively, after PSaV infection in the presence of GCDCA or 22- and 9-fold, respectively, in the absence of GCDCA at 30 mpi, relative to that in mock-infected cells (Fig. 12A and B). As a positive control, in LLC-PK cells incubated for 10 min with 1.8 mM EGTA, a calcium ion chelator, 24- and 15-fold increases in the paracellular diffusion of FD4 and FD70, respectively, were observed. However, LLC-PK cells pretreated for 1 h with RhoA inhibitor (CT04), ROCK inhibitor (Y27632), or MLC inhibitor (blebbistatin) showed pronounced reductions in the transepithelial leakage of FD4 and FD70 induced by PSaV infection in the presence of GCDCA (15- to 16-fold decrease in FD4 permeability or 4- to 5-fold decrease in FD70 permeability) or in the absence of GCDCA (13- to 14-fold decrease in FD4 permeability or 3- to 4-fold decrease in FD70 permeability) (Fig. 12A and B).

TJs work as a fence and prevent the diffusion of molecules from the apical domain to the lateral domains (30, 43). The fence function of the TJ could be evaluated by the lateral diffusion of apically administered fluorescent lipids (BodipyFL-C_12_-sphingomyelin-bovine serum albumin [BSA] complex) in monolayers cultured on Transwell filters (19, 30). Therefore, mock-treated or pretreated LLC-PK monolayers grown on Transwell filters were either mock-infected or infected with PSaV for 30 min. The apical compartments were then incubated with BodipyFL-C_12_-sphingomyelin-BSA complex, and the filters were immediately analyzed via confocal microscopy. Mock-treated and mock-infected LLC-PK monolayers retained the fluorescent lipid at the apical membrane and did not show diffusion toward the lateral regions (Fig. 12C and D). In PSaV-infected cells, however, the fluorescent lipid started to diffuse to the lateral plasma membrane (Fig. 12C and D), consistent with our previous results (30). When the cells were pretreated with RhoA inhibitor (CT04), ROCK inhibitor (Y27632), or MLC inhibitor (blebbistatin), the lateral diffusion of the fluorescent lipid was visibly reduced (Fig. 12C and D). Taken together, these results suggested that the early stage of PSaV infection induced disturbance in the gate and fence functions of TJs by contraction of the actomyosin ring through the RhoA/ROCK/MLC signaling pathway and the functions were restored by the inhibition of this signaling pathway.

## DISCUSSION

As receptors or coreceptors, many viruses belonging to at least nine different families, regardless of the nature of their genome (RNA or DNA), enveloped or nonenveloped, target the molecules present in the AJC such as TJs and adherent junctions (29, 44–50). Therefore, the dissociation of the AJC is an essential prerequisite for the binding of viruses to molecules buried in the AJC. The early stage of PSaV infection also induced disruption of the TJs to reach the occludin coreceptor hidden beneath the TJ on the basolateral surface of polarized LLC-PK cells (30). In addition, feline calicivirus (FCV) and Hom-1 vesivirus in the genus *Vesivirus* are known to use JAM-1 to enter cells (51, 52). However, the signaling pathways involved in the dissociation of TJs by members in the *Caliciviridae* family such as PSaV, FCV, and Hom-1 vesivirus have largely remained unknown. Here, we demonstrated that the dissociation of TJs in polarized LLC-PK cells upon early infection of PSaV is mediated by the activation of the RhoA/ROCK/MLC signaling pathway.

In previous studies, we demonstrated that infectious PSaV particles or PSaV virus-like particles (VLPs) dissociate TJs after binding to cell surface carbohydrate receptors α2,3- and α2,6-linked sialic acids on *O*-linked glycoproteins, which allows them to bind to buried occludin in the TJs as a coreceptor (30, 53). However, the underlying signaling pathways involved in the PSaV-induced dissociation of TJs remain unclear. The actin cytoskeleton associates with myosin II, forming a perijunctional actomyosin ring underlying the TJ complex (25, 27, 54, 55). Contraction of the perijunctional actomyosin ring plays a major role in the regulation of TJ dissociation. This mechanism is mediated mainly by the phosphorylation at Ser19 of a 20-kDa MLC protein (25, 27, 54, 55). In the present study, we showed that the PSaV Cowden strain supplemented with or without GCDCA induced MLC phosphorylation at the early stage of infection. This PSaV-induced MLC phosphorylation was found to be activated by the upstream molecule RhoA GTP and its downstream effector molecule ROCK. These data suggested that activation of the RhoA/ROCK/MLC signaling pathway results in contraction of the actomyosin ring and subsequent opening of TJs, allowing viral access to the occludin coreceptor and cell entry, consistent with our previous results (30). We also showed that inhibitors of RhoA or ROCK reduced the activation of pMLC, which in turn blocked the dissociation of PSaV-induced TJs, as evidenced by the restoration of the decreased TER, increased paracellular flux, intracellular translocation of occludin, and lateral membrane lipid diffusion. Our results were further confirmed by a reduction in virus replication (∼0.8- to 1-log-unit reduction in the genome copy number, ∼0.8- to 1.2-log-unit reduction in the infectivity of viral progeny, and 50% reduction in viral protein expression) by these inhibitors. These results are similar to those we reported for cells infected with RVA or incubated with its outer capsid protein VP8*, which activated the RhoA/ROCK/MLC pathway (29). Combining our previous results, we conclude that binding of PSaVs to cell surface carbohydrate receptors activates the RhoA/ROCK/MLC signaling pathway to dissociate TJs and then to allow PSaVs to bind to buried occludin as a coreceptor to enter the cells (30, 53).

The modest effect of chemicals, i.e., RhoA inhibitor (CT04), ROCK inhibitor (Y27632), or MLC inhibitor (blebbistatin), on PSaV replication could be explained on the basis that the inhibitory doses used at optimal concentrations were not sufficient, so that PSaVs could bind to buried occludin, enter, and complete the life cycle in the cells. Alternatively, other signaling pathways or mechanisms, such as cAMP, PKA, and protein kinase G, involved in TJ assembly and disassembly could participate in the dissociation of TJs by PSaV binding to receptors (25). Indeed, similar effects of these inhibitors have been reported in restoration of RVA-induced dissociation of TJs (29). Nevertheless, further studies are required to examine whether other signaling pathways or mechanisms are also involved in PSaV-induced dissociation of TJs.

ROCK can indirectly regulate MLC activity via phosphorylation and inactivation of MYPT, leading to the inhibition of MYPT and thus an increase in MLC phosphorylation (25, 27, 56). In the present study, however, pMYPT levels did not increase, as observed by Western blotting or immunoprecipitation, indicating that pMYPT does not play any role in MLC phosphorylation in PSaV-infected cells. Furthermore, MLC phosphorylation could be activated by the PKC/MLCK signaling pathway (25, 27, 54, 55). However, as in RVA-induced activation of the RhoA/ROCK/MLC signaling pathway (29) and unlike in influenza-induced PKC activation (45), we did not observe any increase in PKC or MLCK activity or decrease in MLC phosphorylation upon treatment with PKC or MLCK inhibitors in PSaV-infected cells. These findings suggest that the PSaV-activated pMLC is not mediated via the PKC/MLCK signaling pathway.

Successful propagation of PSaV and some strains of human norovirus *in vitro* could be achieved by inclusion of bile acids such as GCDCA or intestinal contents in the culture medium (15, 57, 58). We previously demonstrated that inoculation of PSaV particles or VLPs without supplementation of bile acids in the culture medium could dissociate TJs of LLC-PK cells (30), suggesting that, similar to TJ dissociation by interaction between rotavirus VP8* protein and cellular virus attachment factor (19, 29), the binding of PSaV particles to cellular receptors induced dissociation of TJs (30). In the present study, we further demonstrated that inoculation of PSaV particles, even without the supplementation of GCDCA, activated the RhoA/ROCK/MLC signaling pathway, whose corresponding inhibitors reversed PSaV-induced TJ dissociation to almost normal status, including restoration of the decreased TER, increased paracellular flux, intracellular translocation of occludin, and lateral membrane lipid diffusion. Moreover, single inoculation of PSaV particles could not activate the PKC/MLCK and RhoA/ROCK/MYPT signaling pathways. Consistent with our previous findings that binding of PSaV particles or PSaV VLPs to cell surface receptors dissociated TJs (30), we demonstrated here that binding of PSaV particles to the cell surface receptors activated the RhoA/ROCK/MLC signaling pathway involved in TJ dissociation through contraction of the actomyosin ring. Because the treatment of LLC-PK cells with bile acid alone induces TJ dissociation and enhances TJ dissociation in PSaV-infected LLC-PK cells (30, 59), it could clarify that the translocation and the internalization of occludin into the cytoplasm were higher in infected cells supplemented with GCDCA, relative to infected cells alone, and our ongoing research is to confirm whether bile acids such as GCDCA activate signaling pathways related to TJ dissociation.

Since activation of the RhoA/ROCK pathway induces barrier dysfunction and increases permeability in various diseases (18, 20–24), therapeutic attempts involving the RhoA/ROCK/MLC pathway have been made (34, 60, 61). As described above, PSaV infection induces a decrease in TER and an increase in paracellular permeability at the early stage of virus infection. In particular, PSaV-induced early dissociation of TJs was restored by the RhoA inhibitor CT04, ROCK inhibitor Y27632, and MLC inhibitor blebbistatin. Among the therapeutic candidates aimed at inhibition of the RhoA/ROCK pathway, fasudil hydrochloride (HA-1077), a derivative of isoquinoline and the only clinically approved pharmacological inhibitor of ROCK, has been approved for the treatment of cerebral vasospasm since 1995 (34, 60–62). Treatment with fasudil is effective in improving lipopolysaccharide-induced endothelial barrier dysfunction and upregulation of proinflammatory cytokines (63–66). Therefore, therapeutic approaches aimed at enhancing or restoring the intestinal epithelial barrier function could constitute an integral part of an adjunctive therapy for the reversal of PSaV-induced early disruption and diarrhea.

In summary, we found that PSaV infection induced early activation of the RhoA/ROCK/MLC signaling pathway in polarized LLC-PK cells. This leads to the dissociation of TJs, redistribution of occludin into the cytoplasm, a decrease in TER, an increase in paracellular permeability, and lateral diffusion of the lipids to allow access of virions to occludin, finally resulting in PSaV entry into cells. Our study contributes to understanding intracellular PSaV entry and to developing efficient and affordable therapies against PSaV and other calicivirus infections.

## MATERIALS AND METHODS

### Cells and viruses.

LLC-PK cells obtained from the American Type Culture Collection (ATCC) (Manassas, VA, USA) were grown at 37°C in minimum essential medium (MEM) (Welgene, Daegu, South Korea) supplemented with 10% fetal bovine serum (FBS), 100 U/ml penicillin, and 100 μg/ml streptomycin, in a 5% CO_2_ atmosphere. MDCK cells obtained from the ATCC were grown in Dulbecco's modified Eagle's medium (DMEM) (Welgene) supplemented with 10% FBS, 100 U/ml penicillin, and 100 μg/ml streptomycin.

PSaV Cowden strain was recovered from full-length infectious clone pCV4A (60) and propagated in LLC-PK cells supplemented with 200 μM GCDCA (Sigma-Aldrich, St. Louis, MO, USA), 2.5% FBS, 100 U/ml penicillin, and 100 μg/ml streptomycin (15). The virus was then concentrated by ultracentrifugation. The viral titer was determined by the 50% tissue culture infective dose (TCID_50_) assay as described previously (30) and calculated by the method of Reed and Muench (67). Bovine RVA NCDV (G6P6[1]) strain purchased from ATCC was preactivated with 10 μg/ml crystalized trypsin (catalogue no. 27250-*-*018; Gibco, Fort Worth, TX, USA) and propagated in monkey kidney MA104 cells (ATCC) as described previously (29). The virus titer was determined by a cell culture immunofluorescence assay using monoclonal antibodies (MAbs) specific for RVA VP6 protein and was expressed as fluorescence focus units (FFU) per milliliter.

### Reagents and antibodies.

RhoA inhibitor (CT04) was obtained from Cytoskeleton Inc. (Denver, CO, USA), and ROCK inhibitor (Y27632), MLC inhibitor (blebbistatin), MLCK inhibitor (ML7), and PKC inhibitor (Gö 6983) were purchased from Sigma-Aldrich. SlowFade Gold antifade reagent with 4′,6-diamidino-2-phenylindole (DAPI) was obtained from Molecular Probes (Bedford, MA, USA). Specific rabbit polyclonal antibodies against pMLC (Ser19), MYPT, and pMYPT (at Thr853) and rabbit MAbs against MLC2, pan-pPKC, and ROCK were purchased from Cell Signaling (Beverly, MA, USA). Rabbit anti-MLCK MAb was obtained from Abcam (Cambridge, MA, USA). Mouse antioccludin MAb was purchased from Invitrogen (Carlsbad, CA, USA); rabbit anti-claudin and anti-JAM polyclonal antibodies and rabbit anti-ZO-1 polyclonal antibody were obtained from Life Technologies (Eugene, OR, USA). Mouse anti-glyceraldehyde 3-phosphate dehydrogenase (GAPDH) (0411) MAb was obtained from Santa Cruz Biotechnology Inc. (Dallas, TX, USA). Secondary antibodies included horseradish peroxidase (HRP)-conjugated goat anti-rabbit IgG (Cell Signaling), HRP-conjugated goat anti-mouse IgG (Santa Cruz Biotechnology), and AF488-conjugated donkey anti-rabbit IgG and AF488-conjugated goat anti-mouse IgG (Life Technologies).

### Cytotoxicity assay.

Gö 6983 and blebbistatin were dissolved in dimethyl sulfoxide (DMSO), and CT04, Y27632, and ML7 were dissolved in double-distilled water to prepare stock solutions. The cytotoxic effects of the chemicals and their solvents were assessed using the MTT assay, as described previously (29). Briefly, monolayers of LLC-PK cells grown in 96-well plates were incubated for 24 h with medium containing different concentrations of various chemicals. After removal of the medium, 200 μl of MTT solution was added to each well and incubated for 4 h at 37°C in a CO_2_ incubator. Afterward, each well was incubated with 150 μl of DMSO and incubated for 10 min at 20°C. The optical density at 570 nm (OD_570_) was read using an enzyme-linked immunosorbent assay (ELISA) reader. The percent cell viability was calculated using the following formula: [(OD_sample_ − OD_blank_)/(OD_control_ − OD_blank_)] × 100. All of the chemicals were used at concentrations that did not change the cell viability. In each experiment, the chemicals were freshly diluted to the desired concentration using medium before being added to the cell monolayers.

### Treatment of cells with chemical inhibitors.

Confluent LLC-PK or MDCK cells grown in 6-well plates or 8-well chamber slides for 4 to 5 days or 5 to 6 days, respectively, were washed twice with phosphate-buffered saline (PBS) (pH 7.4). The cells were either mock treated or pretreated for 1 h at 37°C with the inhibitory chemicals at the following concentrations: CT04, 20 ng/ml and 2 μg/ml; Y27632, 0.1 μM and 20 μM; blebbistatin, 10 μM; Gö 6983, 10 nM and 1 μM; ML7, 0.1 μM and 10 μM. After two washes with PBS, the cells were incubated with PSaV Cowden strain or bovine RVA NCDV strain for the indicated times and then used for assessment of signaling pathways and measurement of virus titers by TCID_50_ assay, genome copy number by reverse transcription (RT)-quantitative PCR (qPCR), and protein expression by Western blotting, as described below.

### Western blot analysis.

Confluent LLC-PK or MDCK monolayers grown in 6-well plates for 4 to 5 days or 5 to 6 days, respectively, were pretreated with or without various inhibitors, mock infected or infected with PSaV Cowden strain or bovine RVA NCDV strain, respectively, and harvested at different time points. The cells were washed three times with cold PBS and lysed for 30 min on ice using cell extraction buffer containing 10 mM Tris-HCl (pH 7.4), 100 mM NaCl, 1 mM EDTA, 1 mM EGTA, 1 mM NaF, 20 mM Na_2_P_2_O_7_, 2 mM Na_3_VO_4_, 1% Triton X-100, 10% glycerol, 0.1% SDS, and 0.5% deoxycholate (Invitrogen) supplemented with protease and phosphatase inhibitors (Roche, Basel, Switzerland). Cell lysates were centrifuged at 12,000 × *g* for 10 min at 4°C. The supernatants were analyzed for total protein content by using a bicinchoninic acid (BCA) protein assay kit (Thermo Fisher Scientific, Waltham, MA, USA). Denatured cell lysates were resolved by SDS-PAGE and transferred to nitrocellulose membranes (GE Healthcare Life Sciences, Piscataway, NJ, USA). The membranes were blocked for 1 h at room temperature with Tris-buffered saline containing 5% skim milk before they were incubated overnight at 4°C with the indicated primary antibodies. HRP-labeled secondary antibodies were added for 1 h at room temperature, and the immunoreactive bands were detected by an enhanced chemiluminescence reaction kit (Dogen, Seoul, South Korea) using the Davinch-K imaging system (Young-Wha Scientific Co., Ltd., Seoul, South Korea).

### RhoA activation assay.

RhoA activation was determined using a RhoA activation assay kit (Cell Biolabs Inc., San Diego, CA, USA) according to the manufacturer’s recommendations. Briefly, LLC-PK or MDCK monolayers were mock infected or infected with PSaV Cowden strain (MOI of 1) or bovine RVA NCDV strain (MOI of 10), respectively, for the indicated times. The cells were then washed twice with ice-cold PBS (pH 7.2) and lysed in ice-cold 1× lysis buffer (125 mM HEPES [pH 7.5], 750 mM NaCl, 5% Nonidet P-40, 50 mM MgCl_2_, 5 mM EDTA, and 10% glycerol) containing protease inhibitor (Roche). The cell lysates were obtained by centrifugation for 10 min at 14,000 × *g* at 4°C. The supernatants were incubated with Rhotekin RBD agarose beads for 1 h at 4°C for RhoA pulldown. Afterward, the beads were pelleted by centrifugation for 10 s at 14,000 × *g* at 4°C and washed three times with 1× assay buffer. The beads were then resuspended with 2× SDS-PAGE sample buffer, boiled for 5 min, and centrifuged, and the supernatants were subjected to 12% SDS-PAGE followed by Western blot analysis. The separated proteins were immunoblotted with a mouse anti-RhoA MAb.

### Immunoprecipitation assay.

Immunoprecipitation of the target proteins was performed as described previously (29). Briefly, LLC-PK or MDCK cells grown in 6-well plates were infected with or without PSaV Cowden strain (MOI of 1) or bovine RVA NCDV strain (MOI of 10), respectively, and then incubated at 37°C for the indicated time points. Afterward, the cells were washed and lysed as described above. Cell lysates were precleared by incubation with protein A- or protein G-agarose beads for 30 min at 4°C. Subsequently, the precleared cell lysates were incubated overnight at 4°C with antibodies against ROCK or pMYPT. The immune complexes were obtained by incubation with protein A- or protein G-agarose beads for 1 h at 4°C, and the immunoprecipitated proteins were then evaluated by Western blot analysis as described above.

### Confocal microscopy.

LLC-PK or MDCK monolayers grown in 8-well chamber slides pretreated with or without inhibitory chemicals were infected with or without PSaV Cowden strain (MOI of 50) (30) or bovine RVA NCDV strain (MOI of 10) (29), respectively. Virus inocula were removed, and the cells were washed twice with PBS, fixed with −20°C methanol for 5 min, permeabilized by the addition of 0.2% Triton X-100 for 5 min on ice, and washed again with PBS. The chamber slides were then incubated overnight at 4°C with each primary antibody against TJ proteins (1:100 dilution). Subsequently, the cells were washed three times with PBS and incubated with secondary antibodies for 1 h at room temperature. The cells were then washed with PBS containing 0.1% newborn calf serum and mounted with SlowFade Gold antifade reagent containing 1× DAPI solution (Molecular Probes) for staining of the nucleus. Cells were observed using a LSM 800 confocal microscope and analyzed using LSM software (Carl Zeiss, Jena, Germany). The internalized TJ molecules were quantified by measuring the relative fluorescence intensity profiles (in arbitrary units) for each TJ molecule in the cytoplasm, as described previously (29). Briefly, 10 images of cells treated with the aforementioned treatments were obtained using confocal microscopy and were processed and quantified using the ImageJ program (http://rsb.info.nih.gov/ij). Internalization was calculated using the following equation: internalization = (*F*_c_/*F*_total_) × 100%, where *F*_c_ is the relative fluorescence of each TJ molecule in the cytoplasm and *F*_total_ is the total TJ fluorescence. Calculations were based on approximately 50 cells. Internalization of each TJ molecule was expressed as a percentage of the mock-treated, mock-infected control.

### Virus titration using TCID_50_ assay.

Virus titers were determined using the TCID_50_ assay as described previously (29, 30). Mock- or chemical-treated LLC-PK cells in 6-well plates were infected with PSaV Cowden strain at an MOI of 1 for 36 h at 37°C. After three repeated freeze–thaw cycles, 10-fold serial dilutions of clarified virus supernatants were prepared in MEM supplemented with 200 μM GCDCA. Of these dilutions, 200 μl was inoculated to monolayers of LLC-PK cells grown in 96-well plates and incubated at 37°C in a 5% CO_2_ incubator. Virus titers were determined at 6 days postinfection and expressed as TCID_50_ per milliliter values by the method of Reed and Muench (67).

### RT-qPCR.

PSaV genome copy numbers were quantified using RT-qPCR as described previously (29, 30). LLC-PK cells grown in 6-well plates were pretreated with or without the indicated concentration of chemicals. The cells were then infected with the PSaV Cowden strain at an MOI of 1 for 1 h, followed by washing with PBS to remove the unbound viruses. At 36 h postinfection, the infected cell cultures were washed twice with PBS and harvested by three cycles of freezing and thawing, and cell debris was pelleted by centrifugation at 2,469 × *g* for 10 min at 4°C. The supernatants along with the remaining bulk samples were collected and stored at −80°C until use. Total RNA was extracted using an RNeasy kit (Qiagen Korea Ltd., Seoul, South Korea) following the manufacturer’s instructions. The viral genome copy number was determined by one-step SYBR green RT-qPCR using primer pairs specific for viral protein genome-linked (VPg) (29, 30). Each reaction mixture, in a total volume of 20 μl, contained 4 μl of RNA template (1 μg), 10 μl SensiFAST SYBR Lo-ROX one-step mixture (Bioline, Quantace, London, UK), 0.8 μl each of 10 μM forward and reverse primers, 0.2 μl of reverse transcriptase, 0.4 μl of RiboSafe RNase inhibitor, and 3.8 μl of RNase-free water. The amplification conditions used for the VPg primer set consisted of denaturation at 95°C for 10 min, followed by 40 cycles of denaturation at 95°C for 10 s, primer annealing at 60°C for 20 s, and extension at 72°C for 20 s. Tenfold dilutions of a known amount of pCV4A were used to generate the standard curve to calculate the VPg copy numbers in the samples. Each sample was assayed in triplicate on the same qPCR plate in two independent experiments. No-template and no-reverse transcriptase reactions were analyzed routinely as negative controls. Data were collected using Rotor-Gene 6000 software (Corbett Research, Mortlake, Australia).

### Measurement of TER.

The degree of tightness or leakiness of TJs was assessed by measuring the TER as described previously (30). Briefly, polarized LLC-PK cells, grown as monolayers on Transwell PET membrane inserts (catalogue no. 3470; Corning-Costar, Corning, NY, USA), were treated with or without chemicals and infected with or without PSaV Cowden strain (MOI of 50). The TER was measured using the Millicell ERS-2 epithelial volt-ohm meter (Millipore, Bedford, MA, USA), and the electrical resistance in the absence of cells was considered background. The net TER values were calculated by subtracting the resistance of the blank from that of the sample well and multiplying the membrane diameter of the Transwell filter culture plate by the resistance in each sample. All values were given in ohms × cm^2^ (Ωcm^2^).

### Paracellular flux assay.

The permeability of polarized LLC-PK monolayers was determined by measuring the transepithelial passage of FITC-dextran with molecular masses of 4 kDa and 70 kDa (FD4 and FD70, respectively) (Sigma-Aldrich), as described previously (30). Briefly, polarized LLC-PK cells grown on Transwell PET membrane inserts (Corning-Costar) were treated with or without chemicals or infected with or without PSaV Cowden strain (MOI of 50) in the presence or absence of 200 μM GCDCA for 5, 30, or 90 min at 37°C. As a positive control, another set of cells was treated with EGTA (1.8 mM) for 10 min. Afterward, FD4 and FD70 (10 μg/ml) were added to the apical chamber of the Transwell insert, and the cells were incubated for 1 h at 37°C. After the incubation period, the media were collected from the apical and basolateral sides, and the concentration of FITC-dextran was measured using a fluorometer (FluroMax 2; Horiba, Kyoto, Japan) at an excitation wavelength of 492 nm and an emission wavelength of 520 nm.

### Membrane lipid diffusion assay.

Labeling of the cells with fluorescent lipids was performed as described previously (30). Briefly, polarized LLC-PK cells grown on polyester membrane Transwell inserts (catalogue no. 3470; Corning-Costar) were treated with or without chemicals and infected with or without PSaV Cowden strain (MOI of 50) in the presence or absence of 200 μM GCDCA for 30 min. Afterward, they were labeled on the apical side for 10 min on ice with a 5-nmol/ml solution of the BodipyFL-C_12_-sphingomyelin-BSA complex (Molecular Probes). BodipyFL-C_12_-sphingomyelin-BSA complex was prepared in P buffer (145 mM NaCl, 10 mM HEPES [pH 7.4], 1 mM sodium pyruvate, 10 mM glucose, and 3 mM CaCl_2_). The cells were washed three times with cold P buffer and incubated on ice for 30 min. The filters were then cut from the frame, mounted in P buffer on a glass slide, and covered with a coverslip. The samples were analyzed immediately by z-sectioning with a LSM 800 confocal microscope using LSM software.

### Statistical analyses and software.

Statistical analyses were performed on triplicate experiments using GraphPad Prism software version 5.03 (GraphPad Software Inc., La Jolla, CA, USA) and one-way analysis of variance (ANOVA). *P* values of less than 0.05 were considered statistically significant. Figures were generated using Adobe Photoshop CS3 and Prism 5 software version 5.03.
